# Sex-specific association between prenatal androgenization (second-to-fourth digit length ratio) and frontal brain volumes in adolescents

**DOI:** 10.1007/s00406-022-01515-4

**Published:** 2022-11-30

**Authors:** Bernd Lenz, Sarah Gerhardt, Rafat Boroumand-Jazi, Anna Eichler, Verena Nadine Buchholz, Peter A. Fasching, Johannes Kornhuber, Tobias Banaschewski, Herta Flor, Stella Guldner, Maren Prignitz, Frauke Nees, IMAC-Mind Consortium

**Affiliations:** 1 Department of Addictive Behavior and Addiction Medicine, Central Institute of Mental Health (CIMH), Medical Faculty Mannheim, Heidelberg University, Mannheim, Germany; 2 Department of Psychiatry and Psychotherapy, Friedrich-Alexander-Universität Erlangen-Nürnberg (FAU), Erlangen, Germany; 3 Department of Child and Adolescent Mental Health, Friedrich-Alexander-Universität Erlangen-Nürnberg (FAU), Erlangen, Germany; 4 Department of Obstetrics and Gynecology, Friedrich-Alexander-Universität Erlangen-Nürnberg (FAU), Erlangen, Germany; 5 Department of Child and Adolescent Psychiatry and Psychotherapy, Central Institute of Mental Health (CIMH), Medical Faculty Mannheim, Heidelberg University, Mannheim, Germany; 6 Institute of Cognitive and Clinical Neuroscience, Central Institute of Mental Health (CIMH), Medical Faculty Mannheim, Heidelberg University, Mannheim, Germany; 7 Institute of Medical Psychology and Medical Sociology, University Medical Center Schleswig Holstein, Kiel University, Kiel, Germany

**Keywords:** Digit ratio, Anterior cingulate gyrus, Inferior frontal gyrus, Behavioral control, Sex, Gender

## Abstract

Prenatal androgenization associates sex-dependently with behavior and mental health in adolescence and adulthood, including risk-taking, emotionality, substance use, and depression. However, still little is known on how it affects underlying neural correlates, like frontal brain control regions. Thus, we tested whether prenatal androgen load is sex-dependently related to frontal cortex volumes in a sex-balanced adolescent sample. In a cross-sectional magnetic resonance imaging study, we examined 61 adolescents (28 males, 33 females; aged 14 or 16 years) and analyzed associations of frontal brain region volumes with the second-to-fourth digit length ratio (2D:4D), an established marker for prenatal androgenization, using voxel-based morphometry in a region-of-interest approach. Lower 2D:4D (indicative of higher prenatal androgen load) correlated significantly with smaller volumes of the right anterior cingulate cortex (r-ACC; *β* = 0.45) in male adolescents and with larger volumes of the left inferior frontal gyrus orbital part (l-IFGorb; *β* = – 0.38) in female adolescents. The regression slopes of 2D:4D on the r-ACC also differed significantly between males and females. The study provides novel evidence that prenatal androgenization may influence the development of the frontal brain in a sex- and frontal brain region-specific manner. These effects might contribute to the well-known sex differences in risk-taking, emotionality, substance use, and depression. Future research is needed to elucidate the role of prenatal androgenization within the biopsychosocial model.

**Supplementary Information:**

The online version contains supplementary material available at 10.1007/s00406-022-01515-4.

## Introduction

The prenatal window is a sensitive developmental period, during which the exposure to sex hormones organizes the brain with lasting neurobiological and behavioral effects. Animal experiments have established causal evidence that prenatal exposure to sex hormones influences sex-typical behavior, brain structures, and gene expression with effects that persist into adulthood [1–3]. In humans, androgenization during the prenatal development is thought to sex-specifically shape adult behaviors such as risk-taking, aggression [4–6], sociability [7], reduced impulse control [8], and emotional instability [9]. It was also suggested to influence regional brain morphology [10], enhanced reactivity to positive relative to negatively valenced facial cues [11] in young males, and P2a response to motivational stimuli in a predominantly female cohort [12]. Moreover, prenatal exposure to androgens further interacts sex-specifically with the risk for and symptoms of mental illnesses in adolescence and adulthood [13], including addictive disorders [14–16], suicidal behaviors [17], depression [18, 19], and eating disorders [20–22]. On the brain level, this could be particularly related to the structure and function of the prefrontal cortex, which plays a central role in cognitive control, modulates these behaviors, and associates with mental illness symptoms [23–28]. These relationships are also subject to sex differences [29].

However, there is only little knowledge on how prenatal androgen load shapes human frontal brain structure during developmental sensitive periods like adolescence [30]. Here, frontal brain regions are important for top-down cognitive control, and the temporal dissociation of the development of frontal and subcortical brain regions [31] promotes higher risk-taking behavior and stronger sensation seeking. These behaviors increase the risk for substance abuse [32], attention-deficit/hyperactivity disorder [33], suicidal behaviors [17], and depressive disorders [34]. Moreover, the incidence rates of these disorders typically peak during adolescence [35].

To investigate the effects of prenatal androgen exposure on behavioral phenotypes and brain structure, research has widely used the second-to-fourth digit length ratio (2D:4D) as an easily accessible proxy for prenatal androgen exposure [36]. Lower 2D:4D indicates higher prenatal androgenization. Males have lower 2D:4D than females [37], the fetal amniotic testosterone / estradiol ratio correlates negatively with the children’s 2D:4D at the age of two years [38], and higher maternal plasma testosterone collected at amniocentesis associates with lower 2D:4D in the newborn infants [39]. Moreover, it is assumed that 2D:4D is established during the first trimester and changes only little afterwards [40, 41] (but see also [42–45]). 2D:4D does not significantly correlate with peripheral sex hormone levels in adulthood and should thus be independent from direct androgen effects [46]. Some evidence suggests that right-hand 2D:4D (R2D:4D) might be a better marker for prenatal androgenization than left-hand 2D:4D (L2D:4D) [37] and that R2D:4D and L2D:4D are oppositely associated with handedness [47]. The validity of 2D:4D as a marker of prenatal androgen effects is further underlined by experimental rodent studies [48, 49] (but see also [50]) and human data based on conditions with altered prenatal androgen exposure such as congenital adrenal hyperplasia [51], Klinefelter’s syndrome [52, 53], androgen insensitivity syndrome [54], and the twin testosterone transfer [55]. The 2D:4D therefore is thought to give specific insight into the prenatal sex steroid milieu.

It is also important to note that research has established sex-specific associations of 2D:4D with risk for, symptoms of, and severity of mental disorders [13]. For example, a recent meta-analysis supports lower 2D:4D in substance-related and non-substance-related addictions with stronger effects in males than in females [14]. Supporting translational evidence established that in male mice the prenatal androgen receptor antagonism with flutamide decreases alcohol intake of the adult animals, whereas in female mice prenatal androgen treatment increases later alcohol intake. These prenatal androgen receptor modulations also cause differences in expression of genes relevant to addictive behaviors in the adult rodent brain [3]. Moreover, externalizing symptoms [56], aggression [6], and suicide [57] have been related to lower 2D:4D in males, but not in females, and higher 2D:4D has been associated with risk for and symptom severity of depression in females (but not in males) [18, 19] and bulimia nervosa in females [20, 21].

In summary, there is growing evidence for a sex-specific impact of prenatal androgenization (assessed via 2D:4D) on human behavior and mental health. Thus, 2D:4D should also sex-specifically associate with brain function and structure. Previously, Kallai et al. [58] found that lower (i.e., prenatally androgenized) 2D:4D is related to smaller posterior and larger middle hippocampus volumes of the left side. However, the sample consisted of healthy adult females and frontal regions were not the targeted brain areas. We lack knowledge on how prenatal androgenization associates with frontal brain volumes, which are related to behavioral control in male and female adolescents.

## Study aims

In this cross-sectional study, we tested whether the mean of right-hand and left-hand 2D:4D (M2D:4D) relates to brain volumes of frontal cortex regions in males and females aged 14 or 16 years. Because of the previously demonstrated sex-dependent effects, we conducted sex-separated analyses in a first step. Then, we investigated whether R2D:4D is superior to L2D:4D in the statistical models and how 2D:4D and sex interact to influence the brain volumes. Problematic alcohol use is one of the main previously reported risk factors related to 2D:4D. Thus, we were also interested in whether the observed relations between 2D:4D and frontal brain volumes might be a consequence of alcohol consumption, and tested in sensitivity analyses whether alcohol use patterns affect the observed associations. Here, we did not expect and hence not analyze correlations between 2D:4D and alcohol use per se, as the participants in our sample were rather young and had low Alcohol Use Disorder Identification Test (AUDIT) scores.

## Methods

### Sample

The participants were part of the IMAC-Mind subproject 2 (for details see [59]) and recruited via advertising in regional schools and social networks as well as via the registration office of Mannheim, Germany. Inclusion criteria for participation were fluency in speaking German, no psychological or acute / chronic physical diseases, and no medication use. We excluded non-right-handed individuals because of evidence suggesting a relationship between handedness and 2D:4D [47, 60]. For alcohol use no in- / exclusion criteria were applied. Overall, 75 participants aged 14 or 16 years were enrolled. We grouped the participants into females and males according to their biological sex. There was no transgender person in our sample.

The cross-sectional study was approved by the ethical review committee II of the Medical Faculty Mannheim Heidelberg University. Participants were contacted by letter and informed about the study. After telephone screening for inclusion criteria, given detailed study information, and written informed consent of primary caregivers and adolescents, participants were invited to the Central Institute of Mental Health (CIMH) Mannheim, where MRI and 2D:4D measurements were done as part of a larger test battery. Additionally, questionnaires, including the AUDIT [61], were done at home via the online platform SoSci Survey [62]. The measurements and questionnaires for the present project took about 45 min.

### Second-to-fourth digit length ratio (2D:4D)

We scanned the participants’ right and left hands using an Epson Perfection V370 Photo scanner in gray level with 300 DPI resolution. The participants were instructed to remove all jewelry from their hands, slightly spread the fingers, and have contact to the scanner with every finger segment. We used the GNU Image Manipulation Program (GIMP; www.gimp.org) to quantify the length of the second (2D) and fourths (4D) digits, i.e. distance from the middle of the basal crease to the tip of the fingers. Three independent raters (RBJ, BA, AS) measured each finger three times (nine times in total) and were uninformed about sex, age, and brain volumes. We defined M2D:4D as our primary predictor. R2D:4D and L2D:4D were also tested as further predictors. The inter-rater reliabilities (two-way random inter-rater correlation coefficient; absolute agreement) were very high: M2D:4D: *n* = 61, 0.969; R2D:4D: *n* = 61, 0.956; L2D:4D: *n* = 61, 0.963.

### Structural MRI

#### MRI image acquisition and preprocessing

T1-weighted anatomical images were acquired on a 3-T Siemens PRISMA Scanner at the CIMH Mannheim using a 64 channel head coil and an MPRAGE (Magnetization-Prepared Rapid-Gradient Echo) Sequence with 208 slices, TR = 1800 ms, FOV = 250 mm, sagittal orientation, slice thickness = 0.85 mm, Flip angle = 8 degrees, GRAPPA acceleration factor = 3, matrix = 256 × 256 mm and 0.9 × 0.9 × 0.9 mm isometric voxels. Images were preprocessed using the Computational Anatomy Toolbox (CAT12; http://www.neuro.uni-jena.de/cat/) in the Statistical Parametric Mapping software (SPM12; https://www.fil.ion.ucl.ac.uk/spm/) on MATLAB (R2020a; www.mathworks.com). Preprocessing steps included tissue segmentation, spatial registration, bias-correction, and smoothing using a FWHM (Full Width at Half Maximum) 8 mm gaussian kernel. Modulated normalized images were used to extract grey matter volumes in the following ten regions of interest (ROI) in frontal and orbital control areas. These regions are involved in behavioral regulation and mental health, and they are particularly relevant during adolescence [30, 31, 63, 64]: Medial frontal cortex (MFC), medial (MOrG) and anterior (AOrG) orbital gyrus, orbital part of the inferior frontal gyrus (IFGorb), and anterior cingulate cortex (ACC). ROIs were obtained using the neuromorphometrics atlas in CAT12.

### Statistical analyses

#### Voxel-based morphometry ROI analyses

Mean grey matter volumes in each of the ten ROIs for each participant were analyzed in R (https://www.r-project.org/) in the framework of multiple linear regression models for each ROI. Each model included the scaled values of M2D:4D as regressor of interest and age and total intercranial volume (TIV) as nuisance variables. Models were calculated separately for male and female adolescents, as M2D:4D differed significantly between the sexes and to provide evidence separately for males and females. To test how alcohol use affects the observed associations between 2D:4D and frontal brain volumes, we included AUDIT scores as a factor and computed the models again. As it is still debated whether R2D:4D or L2D:4D is superior, we defined M2D:4D as our primary predictor and offer Supplementary Materials showing model outcomes using R2D:4D and L2D:4D. One female participant had to be excluded from these additional analyses due to missing values and two more, because they presented as significant mean-shift outliers based on their studentized residuals in the linear model (Bonferroni-adjusted *p* < 0.05). We also performed additional post hoc linear regression models in ROIs including sex as a factor, where male and female adolescents showed contrasting directions of association to test whether there was a significant dissociation between males and females in these ROIs relative to their individual M2D:4D ratio. That is, we tested whether the regression slopes differed significantly between male and female adolescents in a given ROI. We corrected for multiple comparisons across the number of tested ROIs using the false discovery rate (FDR, [65]). Assumptions for all analyses were tested and met.

## Results

### Sample characteristics and validation hypotheses

Of 75 recruited participants (48.0% female, mean age 15.11 ± 1.00 years), 61 complete datasets (54.1% female, mean age 15.15 ± 1.00 years) were accessible for the present project. Male adolescents differed from female adolescents with significantly lower M2D:4D and R2D:4D, but not L2D:4D (Table 1). Unexpectedly, we also found lower M2D:4D, R2D:4D, and L2D:4D in the participants aged 16 years compared to those aged 14 years (M (SD) [16 years] vs. M (SD) [14 years], *t* (df), *p*; M2D:4D, 0.960 (0.020) vs. 0.977 (0.024), – 3.072 (59), 0.003; R2D:4D, 0.960 (0.024) vs. 0.974 (0.026), – 2.173 (59), 0.034; L2D:4D, 0.959 (0.023) vs. 0.980 (0.026), – 3.317 (59), 0.002). Moreover, female adolescents had generally lower volumes in all ROIs than male adolescents, all *t*s > 2.14, all *p*s < 0.04, with an exception in right IFGorb, where the sexes showed comparable grey matter volumes, mean_males_ = 1.55, mean_females_ = 1.48, *t*(55) = 1.12, *p* = 0.27.

**Table 1:** Sample characteristics

		Male adolescents		Female adolescents		Sex differences
		*N* (%)	*M* (SD)	*N* (%)	*M* (SD)	*t* or χ^2^ (df)
Age	14 years/16 years	12 (42.9)/16 (57.1)		14 (42.4)/19 (57.6)		0.001 (1)
AUDIT score			3.25 (4.6)		3.69 (4.5)	– 0.371 (58)
2D:4D	M2D:4D		0.960 (0.023)		0.973 (0.022)	– 2.267 (59)*
	R2D:4D		0.958 (0.023)		0.972 (0.026)	– 2.140 (59)*
	L2D:4D		0.961 (0.029)		0.974 (0.022)	– 1.914 (59)
Cigarette smoking (at least once during the previous month)	14 years/16 years	0/5		0/3		1.022 (1)
Current activity/job	Student/other	27/1		32/0		1.162 (1)
School type	Middle school^1^	3 (10.7)		2 (6.1)		4.138 (3)
	Comprehensive school^2^	1 (3.6)		4 (12.1)		
	Academic high school^3^	21 (75.0)		26 (78.8)		
	Other	2 (7.1)		0		
Highest graduation of the father	Certificate of secondary education^4^	3 (10.7)		5 (15.2)		4.427 (4)
	Middle School^1^	5 (17.9)		1 (3.0)		
	Qualification for access to higher education^5^	13 (46.4)		14 (42.4)		
	University degree	6 (21.4)		11 (33.3)		
	Other	1 (3.6)		1 (3.0)		
Highest graduation of the mother	Certificate of secondary education^4^	2 (7.1)		1 (3.0)		2.913 (3)
	Middle School^1^	9 (32.1)		6 (18.2)		
	Qualification for access to higher education^5^	13 (46.4)		16 (48.5)		
	University degree	4 (14.3)		9 (27.3)		

*AUDIT* Alcohol Use Disorder Identification Test; 2D:4D, second-to-fourth digit length ratio; R2D:4D, right-hand 2D:4D; L2D:4D, left-hand 2D:4D; M2D:4D, mean of R2D:4D and L2D:4D. * *p* < 0.05

^1^Corresponds to “Realschule“ in German educational system

^2^Corresponds to “Gesamtschule“ in German educational system

^3^Corresponds to “Gymnasium“ in German educational system

^4^Corresponds to “Hauptschulabschluss“ in German educational system

^5^Corresponds to “Abitur/Fachabitur“ in German educational system

### Sex-dependent association between 2D:4D and frontal brain volumes

#### Male adolescents

**Fig. 1:**
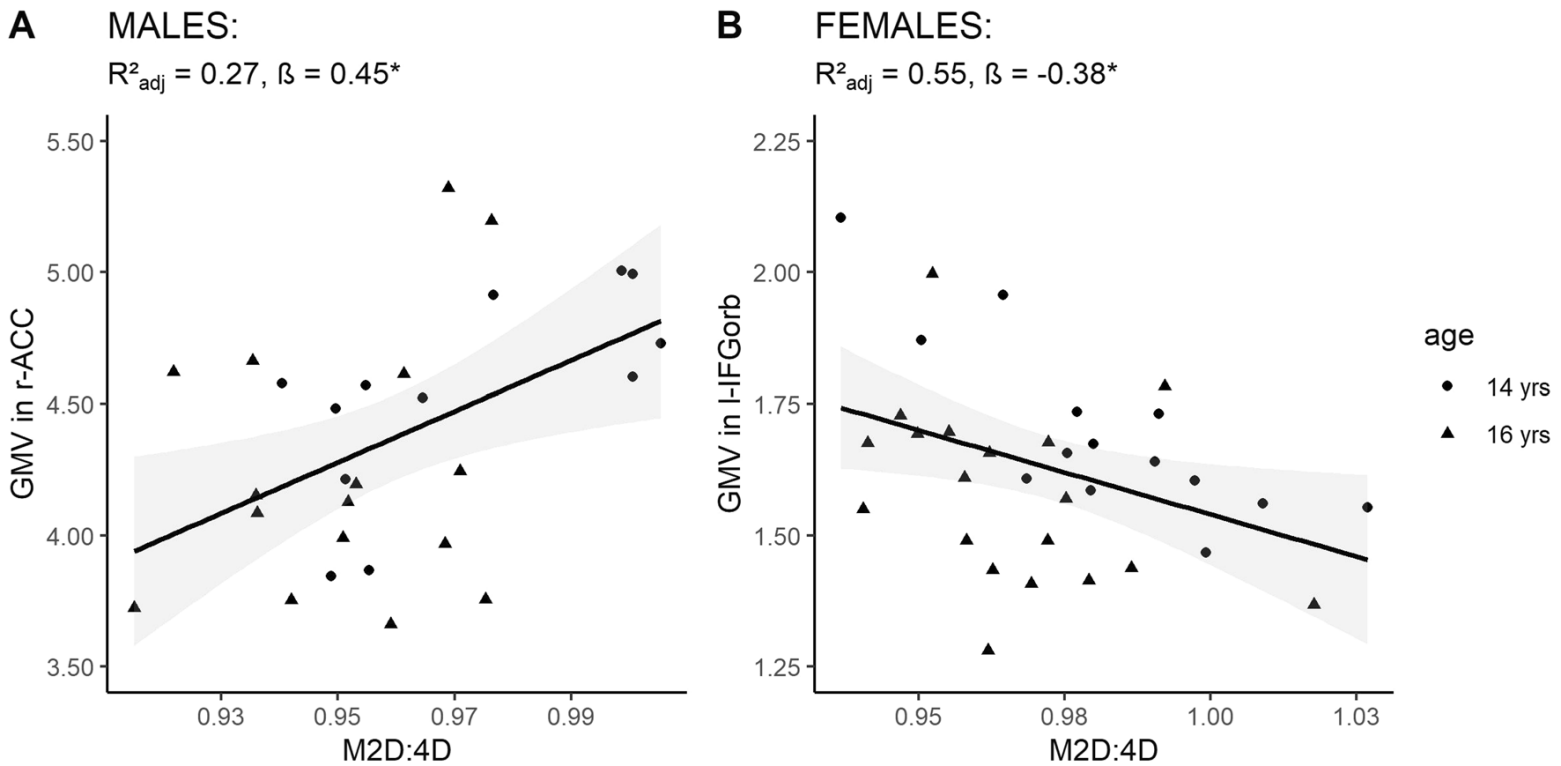
The figures show associations between the mean of right-hand and left-hand second-to-fourth digit length ratio (M2D:4D) and grey matter volumes (GMV) of the right anterior cingulate cortex (r-ACC) in male adolescents (**A**) and the orbital part of the left inferior frontal gyrus (l-IFGorb) in female adolescents (**B**). *p < 0.05. 95% confidence intervals

After correction for multiple hypothesis testing, higher M2D:4D was significantly associated with larger grey matter volumes in right ACC (r-ACC; Fig. 1A). The volumes in left and right mOrG were marginally related to M2D:4D. No further significant associations regarding the other ROIs emerged (Table 2; for covariate contribution to model fits see Supplementary Material 1). The association between M2D:4D and r-ACC volume remained significant after adjustment for AUDIT scores (*F*(4,23) = 3.19, *R*^2^_adj_ = 0.25, *p* < 0.05, *β* = 0.44, *p* < 0.05; see Supplementary Table S1). In separate analyses of R2D:4D and L2D:4D, the observed associations were also present for R2D:4D, but not for L2D:4D (see Supplementary Tables S2 and S3).

**Table 2:** Results from multiple regression models of M2D:4D and male adolescents’ frontal brain region volumes, including age and total intracranial volume as confounding variables

	*β*	Std. Error	*t*	*p*	*F* (3,24)	Adjusted *R*^2^
L Medial frontal cortex	0.00	0.16	0.03	0.98	7.28**	0.41
L Medial orbital gyrus	0.21	0.12	1.84	0.08	22.44**	0.70
L Anterior orbital gyrus	0.08	0.15	0.53	0.60	9.31**	0.48
L Inferior frontal gyrus, orbital part	– 0.01	0.21	– 0.06	0.95	1.25	0.03
L Anterior cingulate cortex	0.23	0.15	1.47	0.15	8.94**	0.47
R Medial frontal cortex	– 0.18	0.15	– 1.21	0.24	9.65**	0.49
R Medial orbital gyrus	0.26	0.13	2.04	0.05^t^	16.53**	0.63
R Anterior orbital gyrus	0.24	0.17	1.39	0.18	5.78**	0.35
R Inferior frontal gyrus, orbital part	0.08	0.17	0.53	0.59	6.72**	0.39
R Anterior cingulate cortex	0.45	0.18	2.49	0.02	4.30*	0.27

Degrees of freedom in parentheses. *p < 0.05; **p < 0.01, ^t^ *p* = 0.051, FDR corrected; M2D:4D, mean of right-hand and left-hand second-to-fourth digit length ratio; *L* left; *R* right; *FDR* false discovery rate

#### Female adolescents

After correction for multiple hypothesis testing, higher M2D:4D was significantly related to smaller grey matter volumes in left IFGorb (l-IFGorb; Fig. 1B). No other ROIs were significantly related to M2D:4D (Table 3; for covariate contribution to model fits see Supplementary Material 2). The association between M2D:4D and l-IFGorb remained significant after adjustment for AUDIT scores (*F*(4,25) = 21.39, *R*^2^_adj_ = 0.74, *p* < 0.001, *β* =  −0.50, *p* < 0.01, see Supplementary Table S4) and was present for both R2D:4D and L2D:4D (see Supplementary Tables S5 and S6).

**Table 3:** Results from multiple regression models of M2D:4D and female adolescents’ frontal brain region volumes, including age and total intracranial volume as confounding variables

	*β*	Std. Error	*t*	*p*	*F* (3,29)	Adjusted *R*^2^
L Medial frontal cortex	– 0.19	0.15	– 1.26	0.22	11.30**	0.49
L Medial orbital gyrus	– 0.08	0.14	– 0.67	0.51	21.20**	0.65
L Anterior orbital gyrus	0.01	0.18	0.47	0.64	4.78*	0.26
L Inferior frontal gyrus, orbital part	– 0.38	0.14	– 2.75	0.01	13.97**	0.55
L Anterior cingulate cortex	0.06	0.13	0.52	0.61	17.01**	0.60
R Medial frontal cortex	– 0.02	0.15	0.18	0.85	12.24**	0.51
R Medial orbital gyrus	– 0.00	0.13	0.06	0.96	16.79**	0.60
R Anterior orbital gyrus	0.18	0.14	1.28	0.21	13.19**	0.53
R Inferior frontal gyrus, orbital part	0.00	0.16	0.06	0.95	9.58**	0.45
R Anterior cingulate cortex	– 0.09	0.16	– 0.60	0.55	8.51**	0.41

Degrees of freedom in parentheses. *p < 0.05; **p < 0.01, FDR corrected; M2D:4D, mean of right-hand and left-hand second-to-fourth digit length ratio; *L* left; *R* right; *FDR* false discovery rate

### Sex-divergent association between 2D:4D and frontal brain volumes

**Fig. 2:**
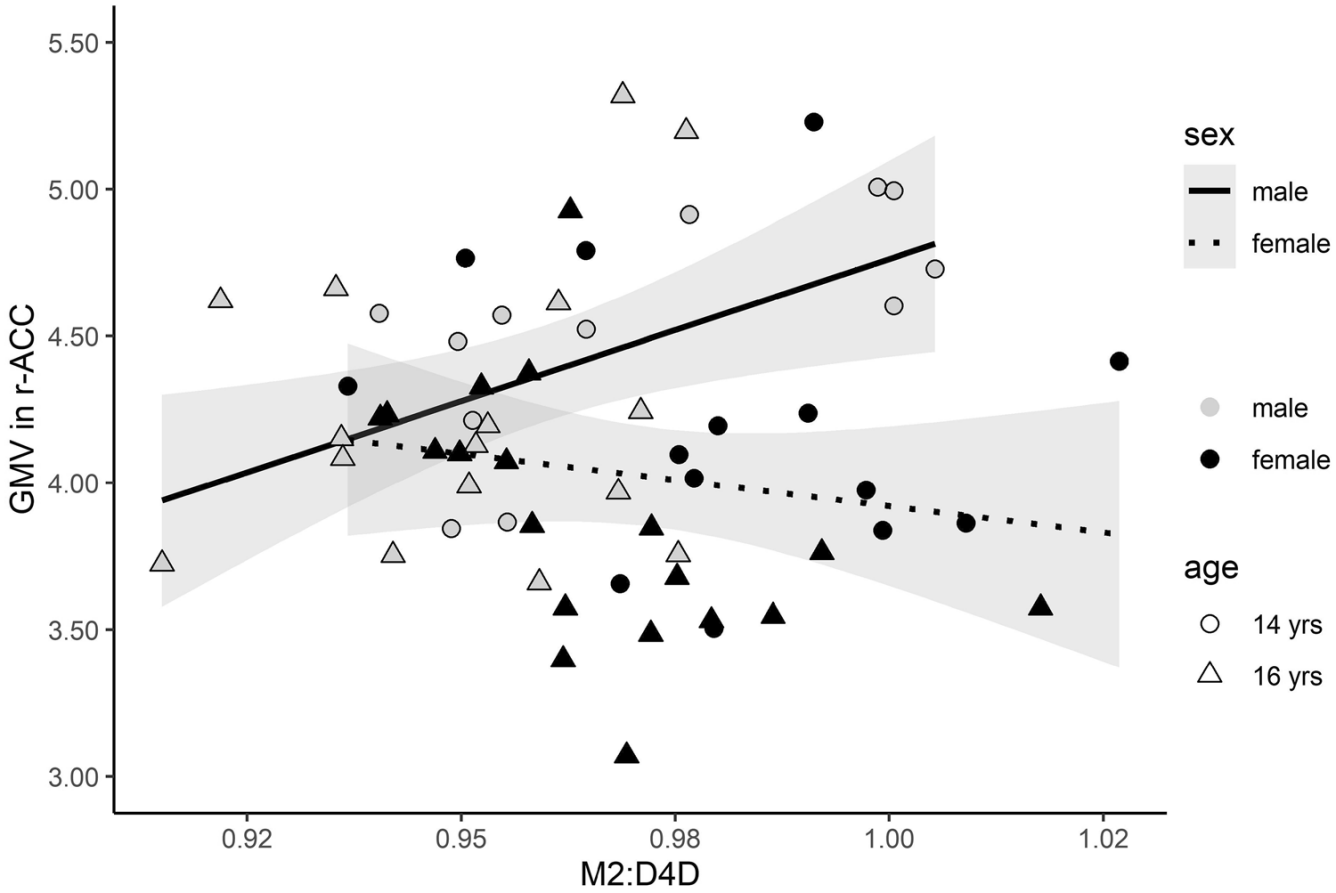
The figure shows sex-separated associations between the mean of right-hand and left-hand second-to-fourth digit length ratio (M2D:4D) and grey matter volumes (GMV) of the right anterior cingulate cortex (r-ACC) (*F*(1,55) = 5.37, *p* < 0.05, male adolescents *B* = 16.46 *vs.* female adolescents *B* = – 4.14, estimate = 20.6, SE = 8.89, *t*(55) = 2.32, *p* < 0.05). 95% confidence intervals

We computed post hoc linear regression models to compare the association between M2D:4D and gray matter volumes in the relevant ROIs for male and female adolescents. There were significant differences between males and females in the relationship between M2D:4D and r-ACC (*F*(1,55) = 5.37, *p* < 0.05, males *B* = 16.46 *vs.* females *B* = – 4.14, estimate = 20.6, SE = 8.89, *t*(55) = 2.32, *p* < 0.05; Fig. 2). This effect persisted after adjustment for AUDIT scores (*F*(1,53) = 4.08, *p* < 0.05, estimate = 18.0, SE = 8.92, *t*(53) = 2.02, *p* < 0.05) and was present in a separate analysis for R2D:4D, but not for L2D:4D (R2D:4D: *F*(1,55) = 12.17, *p* < 0.001, estimate = 27.00, SE = 7.74, *t*(55) = 3.49, *p* < 0.001; L2D:4D: *F*(1,55) = 0.85, *p* = 0.36, estimate = 7.76, SE = 8.40, t(55) = 0.92, *p* = 0.36). The sex difference in the association between M2D:4D and IFGorb volumes between male adolescents, B = -2.51, and female adolescents, *B* = – 17.72, did not reach statistical significance (*F*(1,55) = 2.94, *p* = 0.09).

## Discussion

The prenatal exposure to sex hormones influences the development of the brain with effects that last into adulthood. However, there is a lack of knowledge on how prenatal androgenization shapes brain volumes in developmental sensitive periods. Especially during adolescence, frontal brain regions are important within the context of risky behavior and mental illness. The field is also subject to important sex differences. Hence, we aimed to provide novel evidence for a sex-specific role of prenatal androgenization in frontal brain control regions in an adolescent sample.

In sex-separated analyses, we found that higher prenatal androgen load (indicated by lower 2D:4D) is related to smaller r-ACC in male adolescents and larger l-IFGorb in female adolescents. In post hoc analysis, the ACC associations remained significant for R2D:4D, but not for L2D:4D. This finding is consistent with previous results suggesting that 2D:4D on the right hand might be superior than 2D:4D on the left hand to indicate prenatal androgen exposure [37].

The functioning of the ACC is associated with impulse control, and behavioral inhibition has been associated with right-lateralized prefrontal networks [64]. Thus, the observed association between lower 2D:4D and smaller r-ACC in male adolescents per se and *vs.* female adolescents might indicate that prenatal hyperandrogenization reduces the capacity of behavioral control in males, but not in females. However, it is important to note that the investigated sample consisted of healthy adolescents without known deficits in behavioral control. We also did not directly assess behavioral control in this study, which should be a focus of future research. The ACC and related behavioral control are relevant for addictive behaviors, attention-deficit/hyperactivity disorder, and suicide. Adolescents with less top-down regulation capacity may be more vulnerable to develop substance use disorders [31]. In young alcohol-naive adolescents, those with a high risk for alcohol use disorder due to a positive family history show less inhibitory frontal activation than those with a negative family history [66]. Mashhoon et al. [67] found lower cortical thickness in the right middle ACC of alcohol binge drinkers *vs.* light drinkers. Moreover, the ACC is involved in processing of negative emotions [68], and coping with depressive symptoms is a frequent goal for alcohol use in individuals with alcohol use disorder [69]. Reduced volumes of the ACC are also involved in deficits of impulse control and cognition of patients with attention-deficit/hyperactivity disorder [70]. Furthermore, lower 2D:4D (with the here identified link to frontal brain volumes) has been related to addictions [14, 45, 71, 72], attention-deficit/hyperactivity disorder [13], overactive [8] and externalizing symptoms [56], aggression [4–6], and suicide [17, 57, 73] in males, but not in females. Altogether, these different pieces of evidence might indicate that in males prenatal androgenization organizes frontal brain control regions with lasting reduced behavioral control capacity. Consequently, this might increase the risk to develop mental illnesses, which are more prevalent in males than females particularly regarding addictive disorders (for a review highlighting the complexity of sex differences in substance use disorder see [74]) and attention-deficit/hyperactivity disorder. However, this model certainly needs validation in future studies. In particular, evidence on underlying causality from for example animal models and experimental modulations is needed. It will also be important to investigate whether the here observed associations between 2D:4D and frontal brain volumes are relevant to mental health in later life. Moreover, 2D:4D is related to sociability [7]. Hence, future studies should consider interactions with social and sociocultural aspects. It will be interesting to determine how prenatal androgenization interacts with other biopsychosocial factors (e.g., peer group pressure or self-efficacy expectancy) to associate with behavioral outcomes. In addition, future research should investigate mechanisms that transfer the prenatal influences into adolescence. Epigenetics might be of special interest, as its patterns have been associated with sex hormone activities [75, 76].

Previous research identified alcohol use and misuse as one of the main risk behaviors in relation to 2D:4D [14, 16] and even light-to-moderate alcohol consumption associates negatively with brain volume [77]. Thus, we analyzed whether AUDIT scores affect the here observed associations between 2D:4D and frontal brain structure. The findings remained significant after adjusting the statistical models for the AUDIT scores. Thus, it is unlikely that the observed smaller r-ACC volumes in male adolescents with higher prenatal androgenization is a consequence of alcohol use, but rather might represent a risk factor. The low AUDIT scores in our cohort of underage participants show that most participants did not use alcohol in a hazardous or harmful manner [78], which further supports this assumption.

This study also established in female adolescents an association between higher 2D:4D (indicative of lower prenatal androgenization) and smaller l-IFGorb volumes, a frontal brain region involved in emotion processing [79]. Smaller IFGorb volumes have been found in predominantly female samples of depression [80, 81] and bulimia nervosa [82], and higher 2D:4D has been associated with a higher risk and more severe symptoms of depression [18, 19] (but see also [83]) and bulimia [20, 21] in females. Together with the results observed here, this might indicate that lower prenatal androgen load entails lower l-IFGorb volumes in females with an increased risk for later depressive and eating disorders. However, this assumption needs again further validation in future studies.

The results may have important preventive implications. In combination with additional markers, 2D:4D might evolve as an illness predictor (e.g., the difference between alcohol-dependent patients and controls is of moderate effect size [14]) and thus help to identify individuals who are in particular need for targeted prevention programs. Moreover, human and animal research demonstrated that maternal smoking behavior, alcohol use, and higher stress during pregnancy are related to lower 2D:4D in the offspring [84–86]. Also, early life stress appears to affect neurochemistry within frontal brain areas in a sex-dependent manner [87]. A prospective, controlled, and investigator-blinded study is currently being conducted to test whether the reductions of cigarette smoking, consumption of alcohol, and stress in pregnant women influence 2D:4D in the offspring [88]. Based on the results of our work here, it will be interesting to test whether the aforementioned behavioral intervention during pregnancy is also able to modulate volumes of frontal brain control regions in adolescents with preventive effects. Again, it will be important to study how prenatal androgenization interacts with environmental factors to influence brain structure and function as well as behavior and mental illnesses.

The focus on adolescence as an important developmental period of the frontal brain control areas, the sex-balanced cohort, and the sex-separated analytical approach are important strengths of our study. Moreover, all included participants were right-handed, which is important as some data indicate associations between 2D:4D and hand preference [47, 60]. The limitations of this study include criticism regarding the validity and reliability of 2D:4D [89–91]. Most studies assume that 2D:4D is a marker for the prenatal androgen milieu [90]. However, there is evidence suggesting that estrogens are also involved in the development of 2D:4D. 2D:4D increased after prenatal estradiol treatment in male mice, and it decreased after prenatal estrogen receptor antagonism (fulvestrant) in female mice [49]. Here, we were able to replicate the expected sex differences [37] with lower 2D:4D in male than in female adolescents. Future research should also consider ethnicity/population [41, 92], sexual orientation [93], gender identity [94], and hand preference [47, 60] as potential confounders in 2D:4D research. As expected from the literature [37], we further observed stronger effects on the right hand than on the left hand. In line with previous work using the same method [94–97], the inter-rater agreement for 2D:4D can be interpreted as excellent with inter-rater correlation coefficients greater than 0.950. The here analyzed 2D:4D values are based on hand scans, a method which is more time consuming, but also more precise than using a caliper [98]. We found lower 2D:4D in participants aged 16 years than in those aged 14 years, which was rather unexpected. Previous work established increases in 2D:4D between 20 and 40 months of age (based on hand scans) [44] and from age 1 to age 17 (radiographically determined) [42]. However, serial 2D:4D analysis in the latter study found high reliability for 2D:4D as a trait marker [42]. In rodent experiments, the increase of prenatal testosterone entailed a delayed onset of puberty [99]. Thus, future studies should investigate whether pubertal status influences the observed lower 2D:4D in adolescents aged 16 years than in those aged 14 years. Moreover, we dichotomized the sample into females and males according to the biological sex. Future studies are requested to consider here neglected aspects of the gender concept such as self-defined gender identity and gender expression concerning for example appearance and behavior associated with social norms [100]. The most important limitation is that this study used a cross-sectional design, which does not allow for drawing causal conclusions. Our results suggest that AUDIT scores do not significantly influence the sex-specific associations between 2D:4D and frontal brain volumes. However, future studies should also investigate the effects of other drugs such as cannabis. It is very tempting to infer behavioral consequences from the observed associations between 2D:4D and brain structure. However, brain structure cannot simply be transferred into brain function. To better understand the direct effects of prenatal androgenization on ongoing brain development and behavioral consequences, a longitudinal design and animal experiments will be needed.

## Conclusion

As far as we know, this is the first study to identify that smaller 2D:4D (indicative of higher prenatal androgen load) associates with lower r-ACC volumes in male adolescents and larger l-IFGorb volumes in female adolescents. The results may indicate that the prenatal androgen load affects the development of the frontal brain in a sex- and region-specific and also sex-diverging manner. These brain areas are known to influence behavioral control and they are involved in risk-taking, emotionality, substance use, and depression.

## Supplementary Information

Below is the link to the electronic supplementary material.

Supplementary file1 (DOCX 31 KB)

## Data Availability

The data that support the findings of this study are available from the authors on reasonable request.
