# Supplementary material for: Sex-specific association between prenatal androgenization (second-to-fourth digit length ratio) and frontal brain volumes in adolescents
Source: Eur Arch Psychiatry Clin Neurosci. 2022 Nov 30;273(6):1243–54. doi: 10.1007/s00406-022-01515-4 (PMC10449726; doi:10.1007/s00406-022-01515-4)
Supplement: Supplementary file 1 — Supplementary file1 (DOCX 31 KB) [file 406_2022_1515_MOESM1_ESM.docx]

**Supplementary Material**

**Sex-Specific Association Between Prenatal Androgenization (Second-To-Fourth Digit Length Ratio) And Frontal Brain Volumes in Adolescents**

Bernd Lenz*^,1,2^, Sarah Gerhardt*^,1^, Rafat Boroumand-Jazi^1^, Anna Eichler^3^, Verena Nadine Buchholz^2^, Peter A. Fasching^4^, Johannes Kornhuber^2^, Tobias Banaschewski^5^, Herta Flor^6^, Stella Guldner*^,5,6^, Maren Prignitz*^,6^, Frauke Nees*^,5,6,7^, IMAC-Mind Consortium

^1^Department of Addictive Behavior and Addiction Medicine, Central Institute of Mental Health (CIMH), Medical Faculty Mannheim, Heidelberg University, Mannheim, Germany.

^2^Department of Psychiatry and Psychotherapy, Friedrich-Alexander-Universität Erlangen-Nürnberg (FAU), Erlangen, Germany.

^3^Department of Child and Adolescent Mental Health, Friedrich-Alexander-Universität Erlangen-Nürnberg (FAU), Erlangen, Germany.

^4^Department of Obstetrics and Gynecology, Friedrich-Alexander-Universität Erlangen-Nürnberg (FAU), Erlangen, Germany.

^5^Department of Child and Adolescent Psychiatry and Psychotherapy, Central Institute of Mental Health (CIMH), Medical Faculty Mannheim, Heidelberg University, Mannheim, Germany.

^6^Institute of Cognitive and Clinical Neuroscience, Central Institute of Mental Health (CIMH), Medical Faculty Mannheim, Heidelberg University, Mannheim, Germany.

^7^Institute of Medical Psychology and Medical Sociology, University Medical Center Schleswig Holstein, Kiel University, Kiel, Germany.

*These authors contributed equally to this work.

*Corresponding author at: Department of Addictive Behavior and Addiction Medicine, Central Institute of Mental Health (CIMH), Medical Faculty Mannheim, Heidelberg University, J 5, 68159 Mannheim, Germany. Phone: +49 621 1703-3522, Fax: +49 621 1703-3505. E-Mail: [bernd.lenz@zi-mannheim.de](mailto:bernd.lenz@zi-mannheim.de)

| **Supplementary Material 1.** Exploration of covariate contribution to model fits. For male adolescents, total intracranial volume was associated with grey matter volume in all regions of interest (all βs ≥ .44, all ps ≤ .05), apart from left inferior frontal gyrus, orbital part (β = .28, p = .14) and right anterior cingulate cortex (β = .34, p = .05). Age was associated with grey matter volumes in right medial frontal cortex (β = -.64, p < .05), but no other region (all βs ≥ -.45, all ps ≤ .06). |
| --- |

| **Supplementary Table S1.** Results from multiple regression models of M2D:4D and male adolescents’ frontal brain region volumes, including age, alcohol consumption, and total intracranial volume as confounding variables. | | | | | | |
| --- | --- | --- | --- | --- | --- | --- |
|  | **β** | **Std. Error** | **t** | **p** | **F (4,23)** | **Adjusted R²** |
| L Medial frontal cortex | -.01 | .17 | -.06 | .94 | 5.40** | .39 |
| L Medial orbital gyrus | .20 | .12 | 1.68 | .11 | 16.73** | .70 |
| L Anterior orbital gyrus | .05 | .15 | .35 | .73 | 7.44** | .49 |
| L Inferior frontal gyrus, orbital part | -.02 | .22 | -.09 | .93 | .91 | .01 |
| L Anterior cingulate cortex | .18 | .15 | 1.24 | .22 | 8.65** | .53 |
| R Medial frontal cortex | -.18 | .16 | -1.18 | .25 | 6.94** | .47 |
| R Medial orbital gyrus | .27 | .13 | 2.06 | .05^t^ | 12.1** | .62 |
| R Anterior orbital gyrus | .19 | .17 | 1.16 | .25 | 5.59** | .40 |
| R Inferior frontal gyrus, orbital part | .10 | .17 | .58 | .57 | 4.90** | .37 |
| **R Anterior cingulate cortex** | .44 | .19 | 2.34 | .03 | 3.19* | .25 |
| *Note.* Degrees of freedom in parentheses. *p < .05; **p < .01, ^t^ p = .051, FDR corrected; M2D:4D, mean of right-hand and left-hand second-to-fourth digit lengths ratio; L, left; R, right; FDR, false discovery rate. | | | | | | |

| **Supplementary Table S2.** Results from multiple regression models of R2D:4D and male adolescents’ frontal brain region volumes, including age, alcohol consumption, and total intracranial volume as confounding variables. | | | | | | |
| --- | --- | --- | --- | --- | --- | --- |
|  | **β** | **Std. Error** | **t** | **p** | **F (4,23)** | **Adjusted R²** |
| L Medial frontal cortex | -.06 | .16 | -.41 | .68 | 5.48** | .40 |
| L Medial orbital gyrus | .14 | .11 | -1.19 | .25 | 15.5** | .68 |
| L Anterior orbital gyrus | .12 | .14 | .86 | .40 | 7.79* | .50 |
| L Inferior frontal gyrus, orbital part | .09 | .20 | .46 | .65 | .97 | .00 |
| **L Anterior cingulate cortex** | .29 | .13 | 2.25 | .03 | 10.72** | .59 |
| R Medial frontal cortex | -.15 | .15 | -1.00 | .33 | 6.74** | .46 |
| **R Medial orbital gyrus** | .28 | .12 | 2.30 | .03 | 12.77** | .64 |
| R Anterior orbital gyrus | .19 | .16 | 1.24 | .23 | 5.68** | .41 |
| R Inferior frontal gyrus, orbital part | .20 | .16 | 1.28 | .21 | 5.5** | .40 |
| **R Anterior cingulate cortex** | .56 | .16 | 3.54 | .00 | 5.41** | .40 |
| *Note.* Degrees of freedom in parentheses. *p < .05; **p < .01, FDR corrected; R2D:4D, right-hand second-to-fourth digit lengths ratio; L, left; R, right; FDR, false discovery rate. | | | | | | |

| **Supplementary Table S3.** Results from multiple regression models of L2D:4D and male adolescents’ frontal brain region volumes, including age, alcohol consumption, and total intracranial volume as confounding variables. | | | | | | |
| --- | --- | --- | --- | --- | --- | --- |
|  | **β** | **Std. Error** | **t** | **p** | **F (4,23)** | **Adjusted R²** |
| L Medial frontal cortex | .04 | .17 | .25 | .80 | 5.43** | .40 |
| L Medial orbital gyrus | .19 | .12 | 1.63 | .12 | 16.58** | .70 |
| L Anterior orbital gyrus | -.03 | .15 | -.17 | .86 | 7.39** | .49 |
| L Inferior frontal gyrus, orbital part | -.12 | .21 | -.53 | .60 | 0.99 | .00 |
| L Anterior cingulate cortex | .03 | .15 | .17 | .87 | 7.77** | .50 |
| R Medial frontal cortex | -.16 | .16 | -1.01 | .33 | 6.74** | .46 |
| R Medial orbital gyrus | .18 | .14 | 1.29 | .21 | 10.41** | .58 |
| R Anterior orbital gyrus | .13 | .17 | .79 | .44 | 5.25** | .39 |
| R Inferior frontal gyrus, orbital part | -.03 | .17 | -.15 | .88 | 4.76** | .36 |
| R Anterior cingulate cortex | .19 | .21 | .94 | .36 | 1.75 | .10 |
| *Note.* Degrees of freedom in parentheses. *p < .05; **p < .01, FDR corrected; L2D:4D, left-hand second-to-fourth digit lengths ratio; L, left; R, right; FDR, false discovery rate. | | | | | | |

| **Supplementary Material 2.** Exploration of covariate contribution to model fits. For female adolescents, total intracranial volume was associated with grey matter volume in all regions of interest (all βs ≥ .57, all ps ≤ .003). Age was associated with grey matter volumes in left medial frontal cortex (β = -.33, p < .05), left medial orbital gyrus (β = -.32, p < .05), and left inferior frontal gyrus, orbital part (β = -.37, p < .05), but no other region (all βs ≥ -.25, all ps ≤ .06). |
| --- |

| **Supplementary Table S4.** Results from multiple regression models of M2D:4D in female adolescents’ frontal brain region volumes, including age, alcohol consumption, and total intracranial volume as confounding variables. | | | | | | |
| --- | --- | --- | --- | --- | --- | --- |
|  | **β** | **Std. Error** | **t** | **p** | **F (4,25)** | **Adjusted R²** |
| L Medial frontal cortex | -.17 | .16 | -1.06 | .30 | 5.92** | .40 |
| L Medial orbital gyrus | -.06 | .14 | -.48 | .64 | 10.70** | .57 |
| L Anterior orbital gyrus | .04 | .19 | .19 | .85 | 3.2* | .24 |
| **L Inferior frontal gyrus, orbital part** | -.50 | .11 | -4.58 | <.01 | 21.39** | .74 |
| L Anterior cingulate cortex | .05 | .14 | .38 | .70 | 10.38** | .56 |
| R Medial frontal cortex | .01 | .15 | .07 | .95 | 7.72** | .48 |
| R Medial orbital gyrus | -.00 | .14 | -.01 | .99 | 10.25** | .56 |
| R Anterior orbital gyrus | .15 | .15 | 1.04 | .31 | 9.19** | .53 |
| R Inferior frontal gyrus, orbital part | -.04 | .14 | -.28 | .79 | 10.78** | .57 |
| R Anterior cingulate cortex | -.11 | .16 | -.67 | .51 | 6.41** | .43 |
| *Note.* Degrees of freedom in parentheses. *p < .05; **p < .01, FDR corrected; M2D:4D, mean of right-hand and left-hand second-to-fourth digit lengths ratio; L, left; R, right; FDR, false discovery rate. | | | | | | |

| **Supplementary Table S5.** Results from multiple regression models of R2D:4D in female adolescents’ frontal brain region volumes, including age, alcohol consumption, and total intracranial volume as confounding variables. | | | | | | |
| --- | --- | --- | --- | --- | --- | --- |
|  | **β** | **Std. Error** | **t** | **p** | **F (4,25)** | **Adjusted R²** |
| L Medial frontal cortex | -.26 | .15 | -1.74 | .09 | 6.80** | .44 |
| L Medial orbital gyrus | -.10 | .13 | -.79 | .44 | 10.97** | .58 |
| L Anterior orbital gyrus | -.00 | .18 | -.00 | .99 | 3.22* | .23 |
| **L Inferior frontal gyrus, orbital part** | -.42 | .12 | -3.70 | .001 | 17.02** | .69 |
| L Anterior cingulate cortex | -.04 | .13 | -.32 | .76 | 10.35** | .56 |
| R Medial frontal cortex | -.06 | .14 | -.40 | .69 | 7.80** | .48 |
| R Medial orbital gyrus | .03 | .14 | .25 | .81 | 10.29** | .56 |
| R Anterior orbital gyrus | .18 | .14 | 1.31 | .20 | 9.57** | .54 |
| R Inferior frontal gyrus, orbital part | -.07 | .14 | -.57 | .57 | 10.95** | .58 |
| R Anterior cingulate cortex | -.12 | .15 | -.80 | .43 | 6.50** | .43 |
| *Note.* Degrees of freedom in parentheses. *p < .05; **p < .01, FDR corrected; R2D:4D, right-hand second-to-fourth digit lengths ratio; L, left; R, right; FDR, false discovery rate. | | | | | | |

| **Supplementary Table S6.** Results from multiple regression models of L2D:4D in female adolescents’ frontal brain region volumes, including age, alcohol consumption, and total intracranial volume as confounding variables. | | | | | | |
| --- | --- | --- | --- | --- | --- | --- |
|  | **β** | **Std. Error** | **t** | **p** | **F (4,25)** | **Adjusted R²** |
| L Medial frontal cortex | -.01 | .17 | -.05 | .96 | 5.40** | .38 |
| L Medial orbital gyrus | .00 | .14 | .02 | .98 | 10.55** | .57 |
| L Anterior orbital gyrus | .07 | .19 | .39 | .70 | 3.28* | .24 |
| **L Inferior frontal gyrus, orbital part** | -.46 | .12 | -3.89 | <.001 | 17.90** | .70 |
| L Anterior cingulate cortex | .16 | .13 | 1.19 | .25 | 11.22** | .59 |
| R Medial frontal cortex | .09 | .15 | .64 | .53 | 7.94** | .49 |
| R Medial orbital gyrus | -.05 | .14 | -.33 | .75 | 10.32** | .56 |
| R Anterior orbital gyrus | .07 | .15 | .46 | .65 | 8.68** | .51 |
| R Inferior frontal gyrus, orbital part | .02 | .14 | .15 | .88 | 10.75** | .57 |
| R Anterior cingulate cortex | -.06 | .16 | -.35 | .73 | 6.24** | .42 |
| *Note.* Degrees of freedom in parentheses. *p < .05; **p < .01, FDR corrected; L2D:4D, left-hand second-to-fourth digit lengths ratio; L, left; R, right; FDR, false discovery rate. | | | | | | |
